# Pitfalls in Intracranial Aneurysm Clipping: How to Avoid and How to Get out of Them

**DOI:** 10.3390/jcm14248794

**Published:** 2025-12-12

**Authors:** Lara Brunasso, Biagia La Pira, Rina Di Bonaventura, Carmelo Lucio Sturiale, Enrico Marchese, Giovanni Sabatino, Alessio Albanese

**Affiliations:** 1 Neurosurgery Unit, University Hospital of Parma (Azienda Ospedaliero-Universitaria di Parma), 43126 Parma, Italy; brunassolara@gmail.com; 2 Neurosurgery Unit, San Carlo Borromeo Hospital (Azienda Socio-Sanitaria Territoriale Santi Paolo e Carlo), 20153 Milan, Italy; biagialapira@hotmail.it; 3 Department of Neurosurgery, Fondazione Policlinico Agostino Gemelli IRCCS, 00168 Rome, Italy; rina.dibonaventura@policlinicogemelli.it (R.D.B.); carmelo.sturiale@policlinicogemelli.it (C.L.S.); enrico.marchese@policlinicogemelli.it (E.M.); giovanni.sabatino@policlinicogemelli.it (G.S.)

**Keywords:** intracranial aneurysm, microsurgical clipping, aneurysm surgery, aneurysm complications, intraoperative rupture, brain ischemia, aneurysm remnants, indocyanine-green videoangiography, micro-Doppler flowmetry, aneurysm surgery strategies

## Abstract

**Background/Objectives:** Surgical clipping remains a fundamental treatment modality for intracranial aneurysms, particularly in complex cases and those not amenable to endovascular approaches. However, it is associated with several technical challenges and potential complications that may compromise patient outcomes. This study aims to identify and analyze the most frequent and critical intraoperative pitfalls encountered during microsurgical clipping. We discuss experience-based strategies for avoiding these complications as well as practical solutions for managing them effectively when they occur. **Methods:** A retrospective review of our institutional experience with surgically treated intracranial aneurysms is reported. The study includes a comprehensive analysis of complications encountered across a defined series of cases, along with representative clinical cases. **Results:** Several categories of complications were identified, including aneurysm rupture, incomplete clipping and aneurysm remnant, vessel stenosis and brain ischemia, and new-onset seizures. Specific microsurgical techniques, intraoperative tools (e.g., indocyanine-green angiography, neurophysiological monitoring, micro-Doppler flowmetry evaluation), and decision-making algorithms are discussed to help mitigate these risks. For each scenario, tailored rescue strategies are outlined based on both the literature evidence and our clinical experience. **Conclusions:** Awareness of the potential pitfalls in aneurysm clipping and a structured approach to their prevention and management are crucial for optimizing surgical outcomes, and for preparing young vascular neurosurgeons. Through a combination of technical refinement and scenario-based preparedness, many complications can be anticipated and effectively addressed.

## 1. Introduction

Endovascular advances have reshaped aneurysm care, yet microsurgical clipping remains essential for specific scenarios: complex broad-necked, giant, or fusiform lesions; partially thrombosed aneurysms; bifurcation aneurysms with perforator involvement; and recurrences after endovascular therapy. While randomized and long-term cohort data consistently show the superior durability of complete occlusion and lower retreatment with clipping [1,2,3], parallel population studies document a steady shift toward endovascular treatment and declining open volumes, concentrating surgical exposure in a smaller subset of high-complexity cases [4]. These trends heighten the premium on meticulous operative planning, technical excellence, and reliable intraoperative rescue strategies. For ruptured aneurysms, contemporary guidelines emphasize rapid aneurysm occlusion in comprehensive centers with access to both endovascular and microsurgical options, with a modality selection individualized to the aneurysm morphology, patient condition, and team expertise [5]. For unruptured aneurysms, recommendations similarly endorse case-by-case selection, recognizing the durability of clipping and the minimally invasive appeal of coiling and flow diversion [6]. Within this decision space, surgery often enters precisely where endovascular options are limited—making prevention and management of intraoperative complications a central determinant of outcomes.

Intraoperative adverse events (AEs) remain non-trivial even in high-volume centers. Systematic characterizations report a broad spectrum—premature rupture, arterial or perforator injury, brain swelling, ischemia after temporary clipping, venous outflow compromise, and clip-induced stenosis or neck remnant—with reported intraoperative rupture rates typically in the single- to low-double-digits, higher for ruptured aneurysms and certain locations [7,8]. These realities argue for a “prevention-first, bailout-ready” mindset: secure proximal control, anticipate rupture vectors, preserve venous corridors, protect perforators, and use rehearsed contingency maneuvers. Adjunct technologies improve intraoperative decision-making [9,10], and form the backbone of complication prevention and rescue when integrated with disciplined microdissection and standardized verification checkpoints.

As open aneurysm volumes contract, operative exposure for trainees and structured training have become less frequent, while the technical and cognitive demands of surgery have increased. In this context, maintaining refined microsurgical skills and a rigorous decision-making framework is essential for both ruptured and unruptured aneurysms. Successful aneurysm surgery depends on more than technical dexterity. It requires meticulous anatomical knowledge of the parent vessels, adjacent cranial nerves and perforators, and regional venous anatomy, as well as disciplined preoperative planning. High-fidelity, perfused, and mixed-reality simulators have emerged to maintain skills and shorten learning curves for rare but high-stakes events [11,12,13]. Equally important are a prevention-first mindset in the operating room and mental preparation—developing a reproducible plan of attack, rehearsing critical steps, anticipating failure modes, and cultivating calm execution under stress. While principles such as proximal control, thoughtful arachnoid dissection, and safe clip application are widely taught, the concrete “bailout” strategies that avert or mitigate complications are often learned idiosyncratically rather than systematically.

Building on a 15-year single-surgeon experience, we analyze the most consequential intraoperative pitfalls across key phases—exposure, vascular control, aneurysm dissection, clip application, and verification—and pair each with concrete “how to avoid it” checklists and “how to get out of it” bailout maneuvers, illustrated by representative cases.

## 2. Materials and Methods

We conducted a retrospective observational study of more than 200 consecutive aneurysm clippings performed by the senior author (A.A.) and team over a 5-year period (2020–2024) at the Department of Neurosurgery, Catholic University of the Sacred Heart, “A. Gemelli” Hospital, in Rome. Both ruptured and unruptured intracranial aneurysms treated by open microsurgical clipping were included.

Inclusion criteria were (i) saccular or complex aneurysms of the anterior or posterior circulation; (ii) treatment by open microsurgical clipping; and (iii) availability of complete pre- and postoperative neuroimaging and intraoperative video documentation. Exclusion criteria were pediatric age (<18 years), vascular malformations other than aneurysms, isolated parent-artery occlusion or trapping without clip reconstruction, primary bypass procedures without aneurysm clipping, and cases lacking essential imaging or video records.

Two authors (C.L.S. and R.D.B.) independently reviewed intraoperative videos and imaging using a standardized data-extraction form that cataloged: (1) approach/craniotomy choices; (2) microdissection sequences; (3) clip selection/orientation; (4) verification checkpoints; (5) unintentional technical events; and (6) preventive and rescue maneuvers. Disagreements were resolved by consensus with the senior author (A.A.). Procedures were performed via standard skull base and microsurgical approaches tailored to aneurysm location. Intraoperative verification typically included indocyanine-green videoangiography (ICG-VA) and micro-Doppler flowmetry to assess parent, branch, and perforator patency. Selective early DSA (24–72 h) is obtained when exposure constraints or intraoperative findings raise concern for remnant, stenosis, or perforator compromise, or when a protective residual is intentionally left for staged endovascular completion. In routine concordant ICG/Doppler cases, CTA is preferred over MRA for clip surveillance.

For each case, we abstracted demographics, rupture status, aneurysm location and morphology, surgical approach and craniotomy, proximal/distal control strategy, use and duration of temporary clipping, clip configuration, and adjunct tools. Pre- and postoperative imaging (CTA/DSA/MRI/MRA) and operative videos were reviewed to identify intraoperative AEs and corresponding preventive or rescue maneuvers. Early postoperative outcomes included completeness of occlusion and presence of ischemic or hemorrhagic complications on imaging; neurological status at discharge was recorded where available.

This technical report and the operative “tips and tricks” it presents are grounded in the cumulative experience of 220 surgically treated intracranial aneurysms (100%) at our institution over a 5-year period. Of these, 27 aneurysms (12.2%) were ruptured, and 193 (87.8%) were unruptured at presentation. Although the primary aim of this article is technical and educational rather than a formal cohort-based outcome analysis, we provide a descriptive overview of the underlying case series to contextualize the recommendations. Intraoperative rupture occurred in approximately 15% of ruptured aneurysms and 2% of unruptured aneurysms. Postoperative brain ischemia was documented as asymptomatic in about 25% of ruptured and 5% of unruptured cases, and as symptomatic in 10% of ruptured and 1.6% of unruptured aneurysms. Cranial nerve deficits were mainly observed for the olfactory nerve (CN I), with postoperative impairment in roughly 10% of unruptured cases treated via a subfrontal approach and 25% of ruptured cases, whereas CN II and CN III deficits were not observed in either group; lower cranial nerve dysfunction was absent in unruptured cases and not reliably assessable in ruptured cases due to the severity and complexity of the acute presentation. Residual aneurysm (remnant) was identified in about 15% of the entire cohort, of which approximately 10.5% were expected remnants (intentional, strategy-related) and 5.3% were unexpected remnants; the management strategy for remnants was similar in ruptured and unruptured aneurysms. Postoperative seizures occurred in about 1% of unruptured cases, whereas this complication was not systematically assessable in ruptured aneurysms, where seizures and related complications are often intrinsic to the initial hemorrhagic insult and the emergency treatment strategy. Neuropsychological impairment was observed in approximately 2% of unruptured patients in the immediate postoperative period, but no persistent deficits were detected at long-term follow-up (up to 12 months); in ruptured patients, formal neuropsychological assessment was not performed, as cognitive status was tightly linked to the severity of the initial acute condition. Within this framework, the operative advice and bailout strategies described in the manuscript are derived from and illustrated by this 220-case experience, but the article remains intentionally structured as a technical report, focused on intraoperative phenomena and practical surgical decision-making rather than on hypothesis-driven statistical analysis of the caseload.

Based on recurring patterns observed across cases, the team synthesized an educational algorithm that maps each operative phase to specific “how to avoid it” precautions and “how to get out of it” bailout strategies. The algorithm was iteratively refined through operative video conferences and morbidity-and-mortality meetings until consensus was reached.

The Institutional Review Board of our Institution waived the requirement for ethical approval due to the retrospective analysis of de-identified data and the use of anonymized clinical data and operative images for research and educational purposes. Patients at our institution routinely consent—within the standard informed-consent procedure—to the use of de-identified data and images for scientific and didactic purposes, in accordance with applicable data-protection regulations. A statistical analysis is beyond the scope of this article.

## 3. Results

Table 1 presents a practical prevention and bailout checklist to guide intraoperative decision-making when approaching an intracranial aneurysm. As summarized, the pitfall categories, preventive measures (“How to avoid”), bailout strategies (“How to get out of it”), and intraoperative adjuncts are distilled from the published literature and then applied to the operative scenarios encountered in the present series. The table should be read as an evidence-based checklist that contextualizes our experience; bracketed citations inside the table map each item to its source paper(s).

### 3.1. Intraoperative Rupture (IOR)

Intraoperative rupture (IOR): Intraoperative aneurysm rupture is widely regarded as the most dreaded and psychologically demanding complication of aneurysm surgery [7,14,15,16]. It occurred more frequently in ruptured aneurysms—particularly with a higher clinical grade (Hunt–Hess/WFNS ≥ III) [17], small ruptured sacs [17], and in cases requiring evacuation of a large intracranial hematoma (“uncorking” effect) [18]. Among unruptured aneurysms, IOR clustered in lesions demanding extensive sharp dissection (e.g., AComA and MCA aneurysms with dense pial/branch adhesions) [19].

#### 3.1.1. How to Avoid (Prevention)

Use minimal, gentle dissection; favor fine, sharp arachnoid/pial releases over traction; minimal manipulation of vessels [20].

Secure proximal control before dissecting the dome; obtain distal control when feasible (trapping readiness for ICA/posterior circulation) [20]; for ICA aneurysms, plan proximal control preoperatively (CTA to assess cervical/subarachnoid segment; readiness for clinoidectomy or cervical ICA exposure if needed).

Prioritize neck-first dissection; treat dome dissection as the final step whenever anatomy permits [20].

Consider temporary clipping during critical steps to reduce wall tension and improve handling; limit single occlusion periods and allow reperfusion intervals [21].

Choose approach corridors (including subpial/transcortical variants) that minimize force vectors on the dome [22].

Preserve venous corridors; maintain brain relaxation; evacuate hematoma only when essential to exposure to avoid sudden decompression [5].

#### 3.1.2. How to Get out of It (Bailout)

Remain calm, avoid panic.

Clear the field; reduce magnification if helpful; use two robust suctions to localize the rent—avoid blind sweeping; perform precise point suction until the bleeding site is visualized.

Apply proximal control first with temporary clip on parent vessel to arrest inflow; if hemorrhage is torrential, add distal control to enable temporary trapping [21]. Pilot control options (when no temporary clip is yet on): Pilot clip directly on the rent, with or without a cottonoid buttress (as popularized by Spetzler [2]); pilot/constructive neck clip to re-form the sac and regain control.

Choose approach corridors (including subpial/transcortical variants) that minimize force vectors on the dome.

Once control is achieved and the anatomy re-identified, proceed with definitive neck reconstruction using a properly oriented clip; avoid applying clips across any blind spot. After hemostasis, verify with ICG-VA and micro-Doppler to confirm parent/branch/perforator patency and to detect residual filling or clip-induced stenosis [23]; revise as needed. Manage temporary-clip time prudently (short occlusion windows with reperfusion), and reassess for ischemia or swelling before closure.

Supplementary Video S1 illustrates a representative case of ACoA intraoperative aneurysm rupture during subarachnoid perianeurysmal dissection.

### 3.2. Brain Ischemia

Intraoperative or early postoperative brain ischemia resulted from the transient or persistent compromise of parent, branch, or perforator flow due to temporary clipping, clip-induced stenosis/kinking, vessel manipulation, or vasospasm [24]. It occurs most commonly during or immediately after clip application and during periods of temporary occlusion [25]. Events were enriched at bifurcations with dense perforator networks (e.g., AComA/A1–A2, MCA M1–M2, PCoA/ICA, and PICA origin) [26,27,28]. Intraoperative adjuncts are pivotal to prevent cerebral ischemia. Continuous neuromonitoring (MEP/SSEP/EEG) provides early functional alarms during temporary occlusion and after clip application, and can guide clip repositioning or adjustments to temporary occlusion to avert neurological deficits [29,30]; micro-Doppler flowmetry supplies quantitative, vessel-specific signals for parent/branch/perforators [10,31]; and ICG videoangiography verifies patency and excludes clip-induced stenosis under the microscope [23,32]. Used together—with planned reperfusion intervals—these tools enable immediate on-table correction (release of temporary clips, clip reorientation/exchange, topical vasodilators), thereby limiting ischemic injury.

#### 3.2.1. How to Avoid (Prevention)

Preserve flow; favor neck-first strategies and clip reconstructions that maintain parent/branch lumen; protect perforators throughout the dissection.

Short temporary clipping; use brief occlusion windows with planned reperfusion intervals; record occlusion times and coordinate with anesthesia for blood pressure augmentation and brain relaxation.

Avoid vessel manipulation; minimize traction, torsion, and clip repositioning passes; use sharp arachnoid/pial releases rather than blunt traction and select clip geometry (e.g., fenestrated/angled) that prevents branch compression or kinking.

#### 3.2.2. How to Get out of It (Bailout)

Immediate checks: If there is a neuromonitoring alert (MEP/SSEP/EEG) [29] or concern for hypoperfusion, release temporary clips, raise mean arterial pressure, irrigate with warm saline (36–38 °C), and treat focal vasospasm topically.

Verify flow: use micro-Doppler flowmetry to quantify parent/branch/perforator signals and ICG-VA to confirm filling/emptying and rule out clip-induced stenosis or perforator compromise [10,23,33].

Clip revision: If stenosis or branch/perforator incorporation is detected, reopen and re-orient the clip under direct vision (no blind spots), exchange for a shorter or differently angled/fenestrated blade, or add/remove an accessory clip to restore lumen geometry. Reassess and document recovery; repeat ICG-VA and micro-Doppler until baseline signals/patterns return and neuromonitoring recovers; otherwise, escalate (additional release periods, alternative reconstruction, or, if available, intraoperative angiography with endovascular rescue in a hybrid setting).

**Figure 1:**
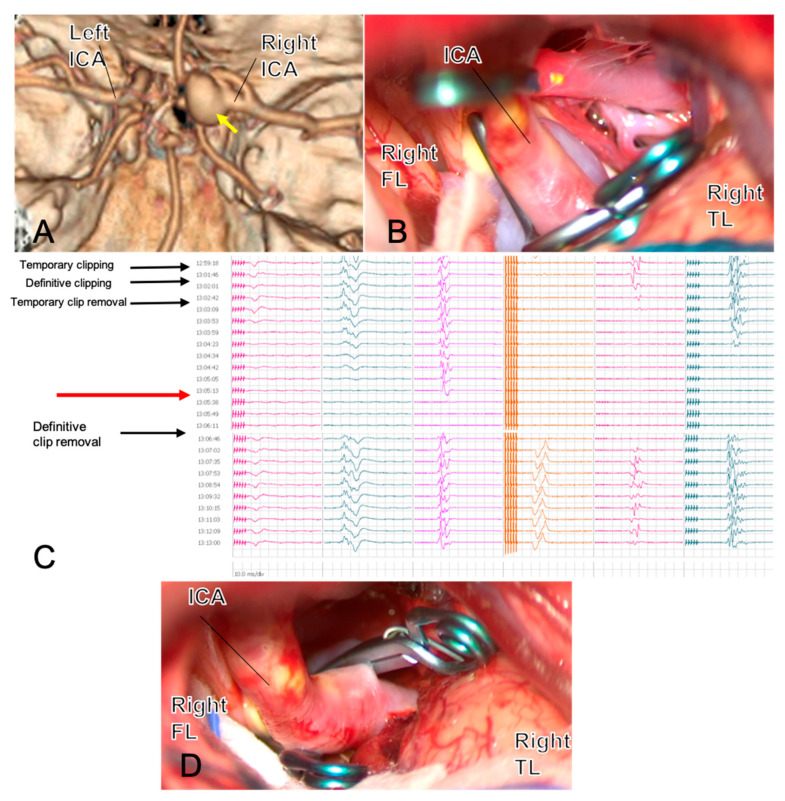
A 54-year-old woman with a large right PCoA aneurysm in fetal PCA configuration ((**A**), yellow arrow) underwent surgical clipping. The first clip across the neck is shown in (**B**). Three minutes after definitive clip closure, MEPs from the left hemisoma dropped to zero ((**C**), red arrow) and recovered immediately after prompt clip removal, indicating flow compromise rather than permanent injury. Review of the 3D reconstruction (**D**) suggested that the aneurysm’s size and posterior–medial orientation created a blind angle potentially harboring an eloquent perforator or the AChA, explaining the neurophysiological change. The clipping strategy was therefore revised: two clips were repositioned on a flow-preserving trajectory, intentionally leaving a small-necked dog-ear to protect efferent/perforator patency, with a plan for staged endovascular completion. After repositioning, MEPs returned to baseline. ICG-VA and micro-Doppler confirmed the patency of parent and branch vessels and complete sac exclusion except for the planned remnant. This case illustrates how intraoperative neuromonitoring can provide real-time feedback that reshapes clip strategy and enhances clipping safety. *Abbreviations: AChA, anterior choroidal artery; FL, frontal lobe; ICA, internal carotid artery; ICG-VA, indocyanine-green video-angiography; MEPs, motor-evoked potentials; PCoA, posterior communicating artery; TL, temporal lobe*.

Figure 1 exemplifies how intraoperative neuromonitoring can redirect clip strategy in real time, transforming an apparently adequate clip into a safer reconstruction and helping prevent brain ischemia in anatomically constrained PCoA (fetal PCA) cases.

### 3.3. Cranial Nerve(s) Deficits

Cranial nerve (CN) morbidity in aneurysm surgery arises from traction, thermal injury, clip-related compression, or ischemia of the nerve or its vascular supply [34]. In our experience, the patterns mirror classic location-specific risks: olfactory dysfunction after frontal base mobilization during anterior communicating artery (AComA) surgery (commonly reported ~10–20% when both frontal lobes are mobilized [35,36]); optic neuropathy with clino-ophthalmic internal carotid artery (ICA) aneurysms (~15%) [37,38]; oculomotor palsy with posterior communicating artery (PCoA) aneurysms (~10%) [39]; and lower cranial nerve deficits (IX–X, XI, XII) during proximal PICA/vertebral artery dissections [40]. These risks are modulated by the operative corridor and by the extent of arachnoid/frontal base manipulation.

#### 3.3.1. How to Avoid (Prevention)

**Figure 2:**
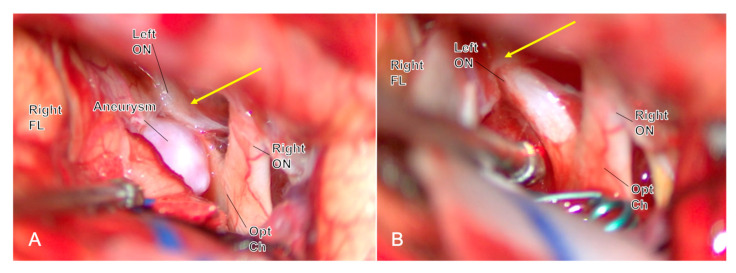
Through a unilateral subfrontal approach during ACoA aneurysm clipping, the arachnoid sleeve over the contralateral optic nerve is intentionally left intact ((**A**), yellow arrow), avoiding bilateral frontal-base mobilization and minimizing the risk of postoperative complete anosmia. After clip application (**B**), the contralateral arachnoid remains ((**B**), yellow arrow) preserved, confirming the olfactory function-preserving strategy. *Abbreviations: ACoA, anterior communicating artery; FL, frontal lobe; ON, optic nerve; Opt Ch, optic chiasm*.

Olfactory nerves (CN I): Prefer a unilateral subfrontal/interhemispheric approach for AComA; avoid routine contralateral subfrontal corridors; maintain the arachnoid adhesion between the frontal lobe and the contralateral optic nerve, keep the frontal lobe on the skull base, and widen the interhemispheric fissure rather than lifting both frontal lobes [41] (Figure 2).

Optic nerve (CN II): For clino-ophthalmic ICA aneurysms, open the falciform ligament early and unroof the optic canal as needed before significant manipulation [34]; protect the nerve with cottonoids; avoid drilling/thermal spread near the canal; gentle nerve mobilization is acceptable after decompression.

Oculomotor nerve (CN III): In PCoA aneurysms, identify CN III early, preserve arachnoid planes around the sac, and plan clip trajectory to avoid nerve impingement; minimize instrument traffic across the cisternal segment [42,43].

**Figure 3:**
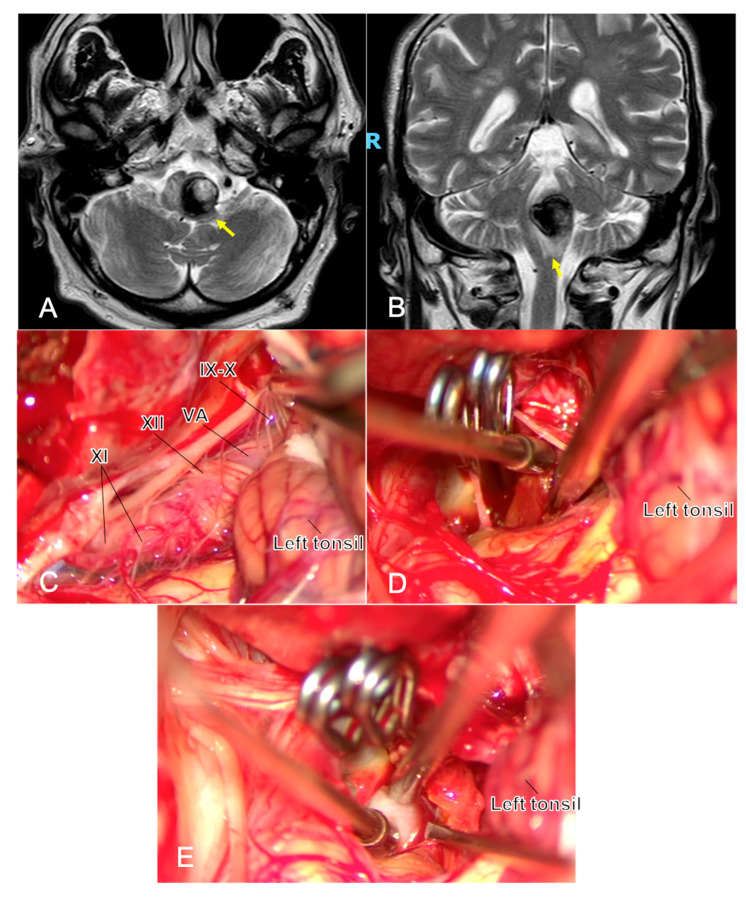
One year after the simple coiling of a previously ruptured p1 PICA aneurysm, a young man developed dysphagia and early right hemiparesis. MRI shows a partially thrombosed recurrent aneurysm at the left VA–PICA junction with marked brainstem compression and surrounding edema ((**A**,**B**), yellow arrows). Microsurgical exposure (**C**) highlights the challenging anatomy at the VA–PICA origin, with the neck arising in close relationship to the lower cranial nerves (IX, X, XI, and XII cranial nerves) and P1 perforators, underscoring the technical difficulty of clip reconstruction in this region. Complete exclusion of the sac is achieved, and the intrasaccular thrombus is evacuated to reduce mass effect (**D**). Throughout the procedure, micro-Doppler flowmetry and neuromonitoring represent two fundamental tools during this clipping, and in (**E**), the application of the micro-Doppler flowmetry is shown to confirm proximal/distal vessel patency and complete aneurysm obliteration. *Abbreviations: MRI—magnetic resonance imaging; PICA, postero-inferior cerebellar artery; VA, vertebral artery*.

Lower cranial nerves (IX–X, XI, XII): During far-lateral/subtonsillar exposure for proximal PICA lesions, proceed with meticulous subarachnoid dissection; avoid suction traction on rootlets; use cranial nerve EMG mapping/stimulation to identify and preserve function, especially after subarachnoid hemorrhage [44] (Figure 3).

#### 3.3.2. How to Get out of It (Bailout)

Immediate protection: If a nerve is at risk or a deficit is suspected intraoperatively, release retraction, irrigate, and allow relaxation; correct blood pressure and avoid brain swelling.

Decompression/revision: CN II: If perfusion or tension on the optic nerve is suspected, further open the falciform ligament and extend optic canal unroofing [45]; revise clip orientation if the ophthalmic/ICA configuration compresses the nerve or its vascular supply. CN III: After clipping a PCoA aneurysm with new ptosis/ophthalmoparesis, free CN III from arachnoid adhesions to the sac, reassess clip blades for contact, and re-orient or exchange the clip to eliminate impingement [42,43]. Lower CNs: With traction-related twitching or mapping changes, relocalize the rootlets using stimulation, adjust the corridor/retractor angle, and continue under direct visualization; confirm PICA patency to avoid ischemic neuropathy [44].

Re-verification: Reassess the field once corrections are made; document nerve integrity (clinical inspection, EMG responses) and ensure hemostasis without re-tethering.

### 3.4. Residual Aneurysm

Residual filling after clipping ranges from a small dog-ear to a substantial neck/dome remnant. Intraoperative remnants arise in two main scenarios: (i) a deliberate choice to preserve flow in an efferent branch or perforator when complete exclusion would jeopardize patency; and (ii) an unexpected residual due to limited visualization or working angles—classically at difficult exposures such as AComA (narrow interhemispheric corridor) and proximal PICA/VA (deep, crowded field) [46,47,48]. The hemorrhagic risk correlates with remnant size and morphology (irregular blebs and enlarging remnants being most concerning), mandating structured verification and follow-up.

#### 3.4.1. How to Avoid (Prevention)

Aim for full circumferential visualization of the neck before definitive clipping; widen the interhemispheric fissure for AComA or the subtonsillar/far-lateral corridor for proximal PICA to eliminate “partial-view” blind spots.

Use neck-first dissection and employ temporary clipping/suction decompression to soften the sac and improve clip placement.

Select clip geometry that preserves branch/perforator flow (e.g., short, angled, fenestrated, tandem reconstruction) and minimizes kinking/stenosis.

Perform routine intraoperative verification—ICG videoangiography and micro-Doppler—to detect dog-ears, neck remnants, or flow compromise early; revise immediately when feasible [23,46].

#### 3.4.2. How to Get out of It (Bailout)

If a remnant is detected and exposure permits, re-orient or exchange the clip; add an accessory clip to flatten a dog-ear or complete neck closure while maintaining lumen geometry [46].

When visualization is the limiting factor, improve the corridor (fissure opening, gentle gyrus rectus/subpial tonsillar expansion, or minor bony work) and reassess under direct vision—avoid closing across blind spots.

If complete exclusion is not safely achievable, perform a protective/partial reconstruction that secures the bleeding point (if ruptured) and preserves efferent flow, with the explicit plan to convert the lesion into an endovascular target (e.g., staged coiling or flow diversion). Document the result with ICG and micro-Doppler; schedule early angiographic follow-up.

Treat enlarging or high-risk remnants endovascularly or with planned re-exploration, depending on anatomy and team resources [49].

### 3.5. Seizures

Early seizures can occur after treatment of ruptured and unruptured aneurysms. Following aneurysmal SAH, the risk is on the order of ~10% and is largely independent of treatment modality (clipping vs. coiling) [50]. In unruptured aneurysms, seizures are less common overall but appear to be relatively more frequent after open surgery than endovascular therapy [50,51,52]. In our series, using minimally invasive corridors—particularly a focused Sylvian split for MCA aneurysms [53,54]—the incidence was negligible, suggesting that cortical exposure/manipulation is a major modifiable driver. Additional contributors include intraparenchymal blood or contusion, clip-related ischemia, hydrocephalus, metabolic derangements (e.g., hyponatremia), and systemic factors (e.g., dialysis).

#### 3.5.1. How to Avoid (Prevention)

Preserve cortex: Small, tailored craniotomy; limited, sharp Sylvian fissure opening; avoid retractor pressure and unnecessary gyrectomy/cautery [53].

Keep the brain “quiet”: Meticulous hemostasis, continuous irrigation to clear subarachnoid/parenchymal blood, normothermia, normoglycemia, and electrolyte control.

Protect flow: Minimize temporary-clip duration; avoid clip-induced stenosis/kinking; treat vasospasm promptly [55].

Selective prophylaxis: For high-risk situations (SAH with intraparenchymal hemorrhage/contusion, temporal lobe dissection, preoperative seizures or epilepsy, large cortical exposure, decompressive procedures), start short-course antiepileptic therapy (e.g., levetiracetam) per institutional protocol; routine prophylaxis is not necessary for low-risk unruptured cases [5].

#### 3.5.2. How to Get out of It (Bailout)

Intraoperative seizure: Irrigate the cortex with cold saline (4–6 °C), stop stimulation, deepen anesthesia (e.g., propofol bolus/infusion), hyperventilate mildly, and correct triggers (bleeding, hypoxia, hypoglycemia, electrolyte imbalance).

Immediate pharmacologic control: Administer a rapid-acting benzodiazepine (e.g., midazolam/lorazepam) followed by antiepileptic loading (e.g., levetiracetam or others per protocol) [5].

Postoperative work-up: Urgent CT/CTA (or CTP when appropriate) to exclude hemorrhage, infarct, or clip-related stenosis; continuous or spot EEG if events recur or if encephalopathy raises concern for non-convulsive status.

Escalation: For persistent seizures/status epilepticus, escalate sequentially (levetiracetam → valproate/lacosamide → anesthetic sedation) per ICU pathway; simultaneously optimize blood pressure, oxygenation, and electrolytes, and treat vasospasm if present.

Follow-through: Continue AEDs short-term (e.g., 1–3 months) when a precipitating brain lesion is present and consider earlier taper in low-risk unruptured cases once imaging is stable and the patient remains seizure-free.

For the ‘Seizures’ category we did not add new static images, as these events are identified primarily through clinical/physiological monitoring (e.g., intraoperative electrocorticography/EEG, anesthetic record, and postoperative EEG) rather than anatomy that a still image could not meaningfully depict. Accordingly, we report recognition criteria, prevention, and management workflows instead of additional panels.

### 3.6. Neuropsychological Impairment

Neuropsychological sequelae after aneurysmal SAH are well recognized and relate chiefly to initial injury burden—clinical grade at presentation, subarachnoid blood volume, early hydrocephalus, and infarction [56,57,58]. In the elective setting, global functional scales (e.g., GOS/mRS) often show no between-modality difference (clipping vs. coiling); however, studies using sensitive testing report subclinical cognitive decline in a substantial proportion of surgically treated patients, particularly early after surgery and in dominant-hemisphere or anterior communicating artery (AComA) cases [59,60,61]. Mechanistic contributors include excessive cortical exposure and retraction, venous congestion from injury to Sylvian/bridging veins, pial insult, and micro-ischemia from temporary clipping or clip-induced stenosis. In our experience—using tailored, minimally invasive corridors with a focused Sylvian split for MCA aneurysms and retractor-free technique [53]—the incidence of clinically relevant impairment was negligible, and this manuscript is currently in preparation.

#### 3.6.1. How to Avoid (Prevention)

Favor less invasive approaches with small, tailored craniotomies and limited, sharp opening of the Sylvian fissure; avoid wide fissure dissection [62].

Use a retractorless technique whenever feasible; if retraction is required, keep it intermittent, low-pressure, and short-duration.

Preserve venous drainage (Sylvian veins, vein of Labbé, bridging veins); alter the corridor rather than sacrificing veins [63].

Maintain brain relaxation (CSF release, osmotherapy as needed, gentle hyperventilation); keep cortex moist and avoid pial contusion/thermal spread.

Protect flow: Brief temporary clipping with reperfusion intervals, clip geometry that prevents kinking/stenosis, and routine ICG-VA and micro-Doppler verification.

In dominant-hemisphere cases, use language-sparing corridors (minimize gyrus rectus resection; respect frontal/temporal opercula) and avoid unnecessary manipulation near eloquent cortex.

#### 3.6.2. How to Get out of It (Bailout)

Intraoperative threat to cognition (swelling, venous discoloration, rising brain tension): Release retraction, irrigate, lower ICP, restore venous outflow (untether veins, reposition cottonoids/retractor), and consider modifying the corridor; if clip-related flow reduction is suspected, revise clip orientation and re-verify perfusion [64].

Postoperative impairment: Obtain urgent CT/MRI to exclude contusion, infarct, hemorrhage, hydrocephalus, or venous congestion; treat vasospasm and hydrocephalus promptly; correct metabolic contributors; initiate early neuropsychological assessment and targeted rehabilitation (speech-language therapy for dominant-hemisphere deficits, cognitive training for executive and memory functions), and optimize sleep, mood, and seizure prophylaxis when indicated.

Overall, careful attention to exposure, venous preservation, brain relaxation, and flow verification appears to reduce not only overt complications but also the subtle neuropsychological changes that sensitive testing can detect after aneurysm surgery.

As before specified for ‘Seizures’, for ‘Neuropsychological Impairment’, no additional images are provided, because these deficits are detected with formal neuropsychological assessment and functional evaluation rather than with illustrative static anatomy. We therefore focus on operative strategies that limit cortical/venous insult and on the postoperative assessment/rehabilitation pathway.

**Table 1:** Prevention and bailout checklist during aneurysm clipping.

Complication	Contexts	“How to Avoid”	“How to Get Out of It”	Intraoperative Adjuncts
**Intraoperative Rupture**	Ruptured; high HH/WFNS; small ruptured sacs; large ICH (“uncorking”); partial view (ACoA, proximal PICA) [17,18,19]	Proximal control first (±distal) [5]; neck-first strategy [20]; gentle sharp dissection [20]; minimal traction [20]; judicious temporary clipping [21]; avoid unnecessary early hematoma evacuation [5]	Keep calm; point suction to localize rent; apply proximal (±distal) control [23]; pilot clip on rent or constructive neck clip [2]; temporary clip on parent; complete reconstruction under direct vision; verify and revise if needed [23]	ICG-VA; micro-Doppler flowmetry; suction decompression
**Brain Ischemia**	Long temporary occlusion [25]; clip-induced stenosis/kink; perforator-rich bifurcations (ACoA, MCA M1-M2, PCoA/ICA, PICA origin) [26,27,28]	Preserve flow (parent/branch/perforators); short occlusion window with reperfusion [21]; avoid vessel manipulation; choose clip geometry that keeps lumen [26,27,28]	Release temporary clips [29]; raise MAP; treat focal spasm; micro-Doppler flowmetry/ICG-VA check [10,23,33]; clip reorientation/exchange or accessory clip until flow restored	Neuromonitoring (MEP/SSEP/EEG); ICG-VA; micro-Doppler flowmetry
**Cranial Nerve Deficit(s)**	CNI: ACoA frontal-base mobilization [35,36]; CNII: clino-ophtalmic ICA [37,38]; CNIII: PCoA [39]; Lower CNs: proximal PICA/VA [40]	Unilateral subfrontal/interhemispheric [41]; preserve olfactory strands [41]; early falciform opening ± optic canal unroofing [34]; plan clip vector away from CNIII [42,43]; meticulous subarachnoid work [42,43]; CN EMG mapping [44]	Release retraction; decompression (extend falciform/canal unroofing) [45]; free nerve from adhesions; revise clip if impinging [42,43]; confirm PICA/VA patency [44]	CN EMG mapping/stimulation; anatomical exploration; navigation
**Remnants**	Intentional flow-preserving partial closure; unexpected residual from limited angle (ACoA, proximal PICA) [46,47,48]	Full neck visualization; widen fissure/corridor; neck-first; appropriate clip geometry; routine ICG-VA/micro-Doppler flowmetry [23,46,47]	Accessory clip or clip exchange; improve exposure (fissure/bone); if unsafe to close completely, protective reconstruction to enable staged endovascular completion; early angiographic follow-up [39,46]	ICG-VA; micro-Doppler flowmetry; early DSA
**Seizures**	SAH (~10% overall, modality-independent); cortical manipulation/contusion; blood burden; metabolic derangement [50,51,52]	Small tailored craniotomy [53]; limited Sylvian split [53,54]; meticulous hemostasis/irrigation [55]; short occlusions; selective short-course AED prophylaxis in high-risk cases [5]	Irrigate [55]; stop stimulation; deepen anesthesia; benzodiazepine + AED loading; CT/CTA; EEG if recurrent; escalate per ICU status protocol [5]	EEG; ICU pathway
**Neuropsychological Impairment**	SAH burden, hydrocephalus, infarct; dominant-hemisphere/anterior circulation; wide exposure; venous injury [56,57,58]	Less-invasive corridors [62]; retractorless technique; preserve Sylvian/bridging veins [63]; brain relaxation; flow verification (ICG-VA/micro-Doppler flowmetry) [23]	Treat hydrocephalus/vasospasm; correct metabolic factors; early neuropsychological assessment and targeted rehab [64]	ICG-VA; micro-Doppler flowmetry; neuropsychological evaluation

Abbreviations: HH, Hunt and Hess scale; WFNS, World Federation of Neurosurgical Societies Scale; ICH, intracranial hematoma; ACoA, anterior communicating artery; PCoA, posterior communicating artery; ICA, internal carotid artery; MCA, middle cerebral artery; PICA, posterior inferior cerebellar artery; VA, vertebral artery; ICG-VA, indocyanine-green videoangiography; MAP, mean arterial pressure; MEP, motor-evoked potentials; SSEP, somatosensory-evoked potentials; EEG, electroencephalography; CN, cranial nerve; EMG, electromyography; SAH, subarachnoid hemorrhage; AED, antiepileptic drug; ICU, intensive care unit; DSA, digital subtraction angiography.

## 4. Discussion

In this single-surgeon, retrospective review, we organized the most consequential complications of aneurysm clipping into a practical, phase-based framework and paired each pitfall with targeted “how to avoid” and “how to get out of it” maneuvers. In keeping with our institutional culture [65,66,67]—where critical self-examination is treated as the primary engine of growth—every surgically treated aneurysm was reviewed to track progressive refinements in approach, including in craniotomy, microsurgical dissection, clip selection/orientation, and the often overlooked “unintentional” technical details. By systematically dissecting intraoperative videos together with pre- and postoperative imaging, we translated these observations—precautions and escape strategies—into lesson-learned rules that could be taught, reproduced, and audited. The resulting checklists—covering exposure/brain relaxation, vascular control, aneurysm dissection, clip application, and verification—are summarized in Table 1 and are intended to function as cognitive aids at the point of care. Items in Table 1 summarize published recommendations that we operationalized within our workflow for the cases described in this manuscript; they are therefore evidence-anchored and experience-applied rather than purely anecdotal.

Evidence supports a volume–outcome relationship for aneurysm care across both endovascular and microsurgical modalities. Current AHA/ASA guidelines for aSAH emphasize management in comprehensive, high-volume centers with 24/7 access to both modalities and dedicated neurocritical care, without mandating a numeric minimum per surgeon [5]. Multiple observational studies define “high-volume” hospitals at around ≥35 aSAH/year, and surgeon experience independently correlates with fewer intraoperative adverse events [68]. In practice, regional hub-and-spoke pathways, transparent monitoring of annual volumes, and adverse-event audits are pragmatic levers to maintain low complication rates as open surgical volumes decline.

Across complications, several cross-cutting themes emerged. First, complication prevention is front-loaded: complete visualization before definitive clipping, neck-first strategies, gentle sharp microdissection, and disciplined control of temporary occlusion times consistently mitigated risk. Second, reliable bailout depends on rehearsed sequences—rapid proximal (and when needed distal) control, field clearing with precise suction, structured clip revision rather than blind clipping, and immediate verification with ICG-VA and micro-Doppler flowmetry. Third, exposure matters: corridors that minimize force vectors on the dome and preserve venous drainage reduced hemorrhagic, ischemic, and neuropsychological sequelae.

Taken together, these observations support three pragmatic messages for contemporary aneurysm surgery:

- Standardize what is standardizable. Checklists (see Table 1), predefined bailout ladders, occlusion-time recording with planned reperfusion, and mandatory IONM/ICG-VA/micro-Doppler flowmetry verification reduce variance and shorten crisis time.
- Design the exposure to lower risk. Corridors that minimize force on the dome, protect veins, and optimize angles for clip control are preventive measures in themselves—not mere preferences.
- Adopt a hybrid strategy for difficult necks. Accepting a protective remnant to safeguard flow—paired with early endovascular completion—can be safer than heroic closure across blind spots.

While the taxonomy presented here is intentionally focused on intraoperative, mechanism-based pitfalls (exposure constraints, clip geometry, temporary occlusion management, and on-table flow verification), we acknowledge that baseline patient factors can modulate overall risk and recovery. Common comorbidities—such as hypertension, diabetes, dyslipidemia, smoking, chronic kidney disease, anticoagulation, and frailty—may increase the likelihood of tissue/vascular fragility, impair cerebrovascular autoregulation, or reduce physiological reserve, thereby narrowing the margin for error when a pitfall occurs. Accordingly, prevention and bailout should be patient-tailored (preoperative optimization, blood-pressure targets, electrolyte/glucose control, careful antithrombotic management, and judicious use of temporary clipping), and multidisciplinary planning is advised in higher-risk patients. A comorbidity-stratified analysis was not undertaken here because it lies outside the educational scope and design of this article; future hypothesis-driven cohort studies are warranted to test how baseline variables interact with the on-table mechanisms described.

Because open volumes are contracting while case complexity increases, deliberate practice must extend beyond the operating room [69,70]. Structured video review, morbidity-and-mortality loops, and simulation (including perfused and mixed-reality models) map well to the phase-based algorithm we propose and can inculcate both prevention habits and bailout fluency in trainees. Embedding the table-based checklists into pre-case briefs and post-case debriefs operationalizes our “prepared-to” philosophy—turning individual experiences into shareable rules that elevate team performance and patient safety.

### Future Directions

Priorities include the following: (i) a prospective, multicenter registry with harmonized adverse event definitions in aneurysm surgery, and time-stamped intraoperative events; (ii) learning-curve analytics (calendar-time and cumulative sum methods) linked to checklist adherence; (iii) routine neuropsychological endpoints in elective cases; and (iv) evaluation of hybrid OR workflows that formalize staged surgical–endovascular completion for planned flow-preserving remnants. These steps would help convert experience-based wisdom into reproducible standards and measurable quality improvements.

In sum, a prevention-first mindset, disciplined verification, and rehearsed bailout sequences—implemented through simple, visible checklists—can make aneurysm clipping safer and more teachable without sacrificing the nuance required for complex cerebrovascular surgery.

## Data Availability

The original contributions presented in this study are included in the article. Further inquiries can be directed to the corresponding author.

## Supplementary Materials

The following supporting information can be downloaded at: https://www.mdpi.com/article/10.3390/jcm14248794/s1, Video S1: During subarachnoid dissection of a ruptured ACoA aneurysm, an intraoperative rupture occurs. Sequential pilot clip attempts are performed under temporary A1 bilateral time-controlled occlusion until the rupture point at the base of the neck is identified and secured. The sequence suggests that manipulation at the neck, further complicated by the aneurysm sac’s characteristic elongated morphology, displaced the rupture point from the adherent clot, reactivating bleeding (still frame, minute 2:11). Bilateral A1 preparation allows rapid, alternating temporary clipping to obtain a clean field, tailor the final clip, preserve flow, and avoid ischemia.

## Abbreviations

The following abbreviations are used in this manuscript:

ACoA	Anterior Communicating Artery
AEs	Adverse Events
AED	Antiepileptic Drugs
CN	Cranial Nerve
CTA	Computed Tomography Angiography
DSA	Digital Subtraction Angiography
EEG	Electroencephalography
EMG	Electromiography
HH	Hunt–Hess Scale
ICA	Internal Carotid Artery
ICG-VA	Indocyanine-Green VideoAngiography
ICH	Intracranial Hematoma
ICU	Intensive Care Unit
MAP	Mean Arterial Pressure
MCA	Middle Cerebral Artery
MEP	Motor-Evoked Potentials
OR	Operating Room
PCoA	Posterior Communicating Artery
PICA	Postero-Inferior Cerebellar Artery
SAH	Subarachnoid Hemorrhage
SSEP	SomatoSensory-Evoked Potentials
VA	Vertebral Artery
WFNS	World Federation of Neurosurgical Societies Scale
